# Broad and Gag-Biased HIV-1 Epitope Repertoires Are Associated with Lower Viral Loads

**DOI:** 10.1371/journal.pone.0001424

**Published:** 2008-01-09

**Authors:** Morgane Rolland, David Heckerman, Wenjie Deng, Christine M. Rousseau, Hoosen Coovadia, Karen Bishop, Philip J. R. Goulder, Bruce D. Walker, Christian Brander, James I. Mullins

**Affiliations:** 1 Department of Microbiology, University of Washington School of Medicine, Seattle, Washington, United States of America; 2 Machine Learning and Applied Statistics Group, Microsoft Research, Redmond, Washington, United States of America; 3 HIV Pathogenesis Programme, Doris Duke Medical Research Institute, University of KwaZulu-Natal, Durban, South Africa; 4 Nuffield Department of Medicine, Oxford University, Oxford, United Kingdom; 5 Partners AIDS Research Center, Massachusetts General Hospital, Harvard Medical School, Charlestown, Massachusetts, United States of America; University of California at San Francisco, United States of America

## Abstract

**Background:**

HLA class-I alleles differ in their ability to control HIV replication through cell-mediated immune responses. No consistent associations have been found between the breadth of Cytotoxic T Lymphocytes (CTL) responses and the control of HIV-1, and it is unknown whether the size or distribution of the viral proteome-wide epitope repertoire, i.e., the intrinsic ability to present fewer, more or specific viral epitopes, could affect clinical markers of disease progression.

**Methodology/Principal Findings:**

We used an epitope prediction model to identify all epitope motifs in a set of 302 HIV-1 full-length proteomes according to each individual's HLA (Human Leukocyte Antigen) genotype. The epitope repertoire, i.e., the number of predicted epitopes per HIV-1 proteome, varied considerably between HLA alleles and thus among individual proteomes. In a subgroup of 270 chronically infected individuals, we found that lower viral loads and higher CD4 counts were associated with a larger predicted epitope repertoire. Additionally, in Gag and Rev only, more epitopes were restricted by alleles associated with low viral loads than by alleles associated with higher viral loads.

**Conclusions/Significance:**

This comprehensive analysis puts forth the epitope repertoire as a mechanistic component of the multi-faceted HIV-specific CTL response. The favorable impact on markers of disease status of the propensity to present more HLA binding peptides and specific proteins gives impetus to vaccine design strategies that seek to elicit responses to a broad array of HIV-1 epitopes, and suggest a particular focus on Gag.

## Introduction

Although attempts to correlate the breadth of the CTL antiviral response and control of HIV-1 infection *in vivo* have been equivocal [1], [2], [3], [4], [5], [6], [7], [8], [9], accumulating evidence support the beneficial role of Gag-specific CTL responses in HIV-1 containment [2], [10], [11], [12], [13], [14], [15], [16], [17], [18] - alluding to differences in antiviral efficacy among specific CD8^+^ T-cell responses. Additionally, certain HLA genes are associated with different rates of disease progression [19], [20], [21], [22], [23], [24], [25], [26], [27], [28], [29]. In particular, HLA alleles B*27 and B*57 seem to confer a survival benefit whereas HLA B*35_Px confers a survival disadvantage [22]. While HLA-B alleles appear to impact disease progression more than other alleles [25], possessing heterologous HLA alleles, and thus possibly allowing the individual to present broader arrays of epitopes, has also been associated with delayed/slower disease progression [24].

The extent of polymorphisms in HIV sequences and in HLA loci underscores that a vast array of HLA/viral peptide combinations can be generated during infection. HIV-1 is characterized by its extensive diversity both among and within infected individuals due to its extreme capacity to mutate, its persistent replication, and the important role of CD8^+^ lymphocyte responses in driving viral evolution [30], [31], [32]. In addition, the HLA region is one of the most polymorphic loci in the human genome. HLA class I molecules are codominantly expressed on antigen-presenting cells such that all six allotypes (if the person expresses heterologous HLA-A, -B and -C proteins) can present viral epitopes.

The fine-mapping of epitopes, typically 9 amino acid (AA) long but ranging from 8 to 11 AA, together with data on their binding properties to HLA molecules, has allowed definition of HLA class I allele-specific sequence motifs that are able to prime virus-specific CD8^+^ T-cell responses. Consistent associations between HLA alleles and disease outcomes suggest an underlying mechanistic function, and prompted us to question whether the scope of the epitope repertoire contributes to the composite effectiveness of the CTL response. “Epitope repertoire” here refers to all viral peptide sequences that fulfill HLA class I allele-specific binding motifs for a specific whole HIV-1 proteome.

Epitope mapping data has been used to develop computational methods of epitope prediction, which are important for the development of diagnostic tools and the design and evaluation of vaccines. Identification of novel HIV-1 epitopes simultaneously fuels our greater understanding of the immune recognition of the HIV proteome and incremental improvements of epitope prediction algorithms [33], [34], [35], [36]. Here, we predicted HLA class I epitopes using a new method based on logistic regression and designed to leverage data across HLA alleles and/or supertypes ([37] available at http://atom.research.microsoft.com/hlabinding/hlabinding.aspx). The prediction method produces approximately 10% false positive results when set to yield 10% false negatives.

We predicted the epitope repertoire in 302 full-length HIV-1 proteomes, isolated from 302 untreated individuals infected with HIV-1 subtype C and B, based on each subject's HLA genotype [32], [38], [39], [40], [41], [42], [43], [44]. We report that a larger epitope repertoire was associated with lower levels of viremia. Furthermore, alleles associated with reduced viral loads tended to target particularly Gag when compared to alleles associated with a lack of control of viral replication.

## Results

### The size of the epitope repertoire differed between HLA alleles and thereby between autologous HIV-1 proteomes

Scanning 302 HIV-1 proteomes, corresponding to 2,718 HIV-1 protein sequences, resulted in the identification of 22,779 epitope motifs, including 8,208 experimentally defined CTL epitopes compiled in databases prior to our study (the latter two figures include redundancies from detection of the same epitopes in multiple individuals). The number of predicted epitopes varied greatly among alleles, i.e., between 1 and 47 per allele per proteome, and henceforth among individuals ranging between 14 and 186 per proteome (mean = 75; median = 72). Given that the vast majority of known epitopes were defined experimentally using peptides corresponding to subtype B, more CTL epitopes are known for subtype B than for subtype C. Thus in turn, more motifs were identified in proteomes from subtype B (mean = 113 per proteome, including 60 previously known epitopes) than in subtype C (mean = 71, including 23 previously known epitopes). To rule out this experimental bias toward HIV-1 subtype B in our study, we verified that the number of epitopes restricted by an allele was not associated with the allele frequency in the population. There were no associations between the number of epitopes restricted by an allele and its frequency in the population in a subgroup of 32 individuals from the Seattle Primary Infection Cohort (r^2^ = 0.0280; p = 0.2307), nor in a subtype C infected South African cohort from Durban (r^2^ = 0.0347; p = 0.1998; n = 270 individuals) or in a representative Sub-Saharan population (r^2^ = 0.0319; p = 0.2197). However, there was a positive relationship between the number of epitopes and the allele frequency in the overall North American population (r^2^ = 0.1153; p = 0.0129; n = 1021 individuals), likely reflecting the focus of HIV/AIDS research on this population. Interestingly, HLA B*27, an allele repeatedly associated with favorable disease outcomes [22] and found 3 times in our dataset, presented the third largest epitope repertoire with 42 predicted epitopes per HIV-1 proteome, i.e., over 3 times the average repertoire size (Mean number of epitopes/HLA allele = 13.87; Confidence Interval (CI) with α = 0.99, Lower CI = 9.46; Upper CI = 18.27).

### Relationship between epitope repertoires and clinical data

**Figure 1:**
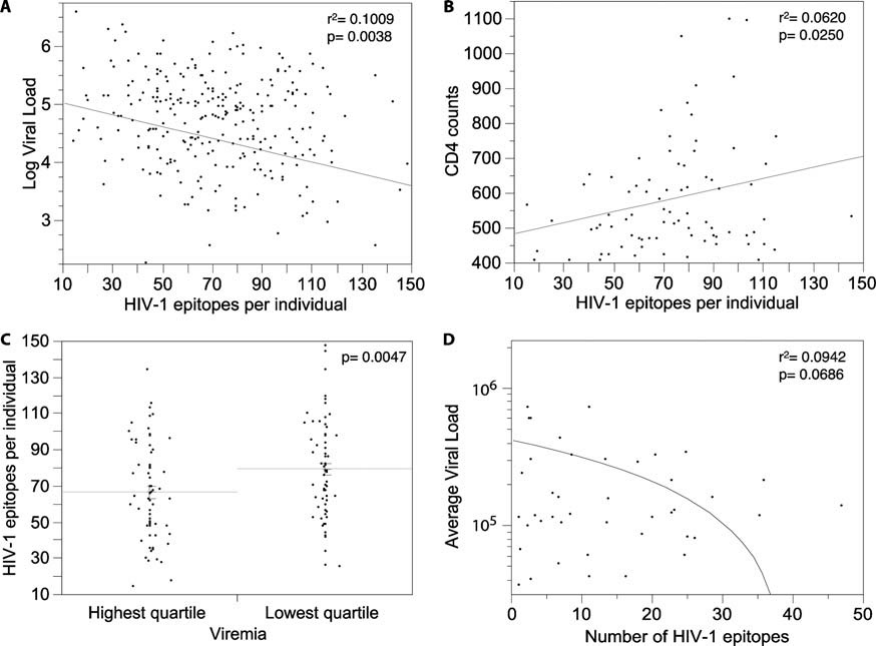
HIV-1 epitope repertoires and clinical data. Putative epitopes were identified *in silico* within full-length autologous HIV-1 proteomes and combined with previously described optimally defined CTL epitopes found in the LANL and IEDB databases. (A) and (B) respectively show the log viral loads and the CD4 counts plotted as a function of the number of predicted HIV-1 epitopes identified per proteome for the individuals with CD4 counts above 400 (n = 81). (C) Shows the number of predicted HIV-1 epitopes for individuals belonging to the highest (mean = 65) and lowest (mean = 76) viremia quartile. (D) Shows the average viral loads of individuals presenting a specific allele as a function of the average number of HIV-1 predicted epitopes for that allele (only alleles presented by at least 3 individuals in the South-African cohort were included).

We compared numbers of predicted epitopes per proteome to viral loads and CD4 counts in subtype C infected individuals from the South African cohort. We found that the more epitopes predicted for an individual, the lower the observed viral load (r^2^ = 0.0446, p = 0.0005; Spearman's correlation factor: Rho = −0.1751, p = 0.0039). In particular, we found a stronger negative relationship between the size of the epitope repertoire and the viral loads among the 81 individuals who had CD4 counts above 400, i.e., when we excluded from the analysis the individuals with vanishing T cell numbers, and presumably function (r^2^ = 0.1009, p = 0.0038; Spearman's correlation factor: Rho = −0.3090, p = 0.0050) (Figure 1A). Additionally, larger epitope repertoires were associated with higher CD4 counts (r^2^ = 0.0620, p = 0.0250; Spearman's Rho = 0.1549, p = 0.0395) (Figure 1B). A relatively weaker association was observed for CD4 than for viral loads, possibly due to CD4 counts being available for only 177 of the 270 individuals evaluated.

By grouping individuals according to their plasma viral loads, we found significantly different numbers of predicted HIV-1 epitopes in individuals within the lowest (<16,437 viral RNA copies/ml; n = 67) and highest (>186,250 copies; n = 67) quartiles. HIV-1 proteomes from individuals in the quartile with the lowest viral load had a mean number of 80 predicted epitopes, compared to 67 in the highest quartile (p =  0.0047) (Figure 1C). There was also a trend for individuals with higher CD4 counts to have more predicted epitopes, the mean number was 76 for individuals in the highest quartile (n = 45; CD4>521.5), and 65 in the lowest quartile (n = 44; CD4<234.5) (p = 0.0686).

The least frequent alleles in the cohort were found to be associated with lower viral loads (Spearman's Rho = 0.2880; p = 0.0448), in agreement with Trachtenberg and colleagues [21]. And, we found a trend indicating that HLA alleles restricting larger repertoires were associated with lower viral loads in HLA-matched individuals (Figure 1D) (Spearman's Rho = −0.2952; p = 0.0517).

### Distribution of epitope repertoires vary between HLA alleles associated with different viral loads

**Figure 2:**
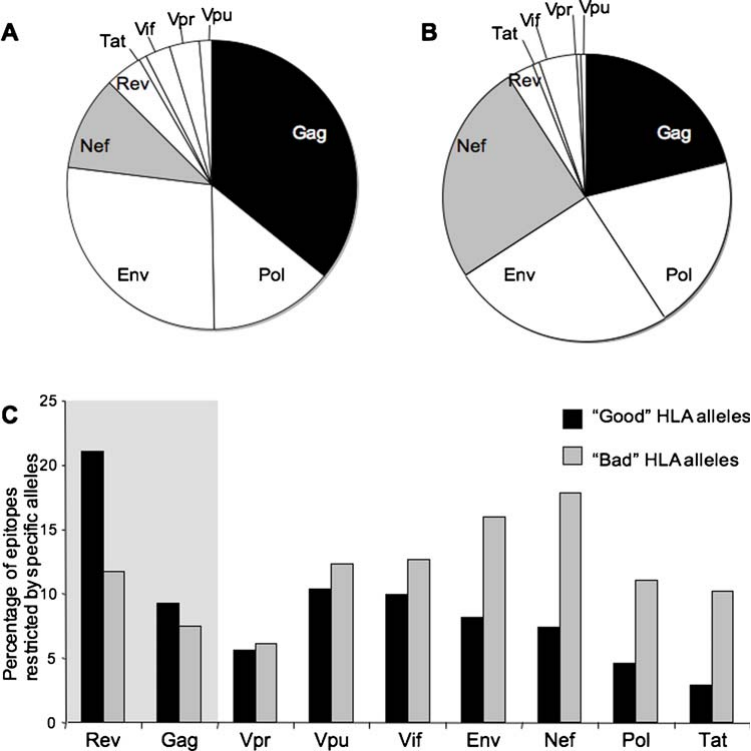
Distribution of epitopes by HLA alleles and by protein. Distribution of epitopes among HIV-1 proteins for HLA alleles associated with lowest/highest viral loads. The ratio of predicted epitopes predicted for each protein corresponded to the number of epitope-fulfilling motifs identified in each protein over the total number of epitopes identified for the whole proteome. (A) Shows the distribution of epitopes for “good” alleles, i.e., those associated with the lowest viral loads in the cohort (lowest quartile: VL<125,437; mean = 65,384; median = 58,229). (B) Shows the epitope distribution for “bad” alleles, those associated with the highest viral loads in the cohort (highest quartile: VL>320,643; mean = 971,587; median = 531,208). For each allele belonging to a quartile, average values per allele were calculated based on the viral loads of HLA-matched individuals). (C) Illustrates the percentage of epitopes restricted by “good” and “bad” HLA alleles for each protein.

Next, we ranked HLA alleles by the average viral loads of subjects in the Durban cohort: the quartile with the lowest viral loads (<125,437 viral copies; mean = 65,384; median = 58,229) included 12 alleles, herein referred as “good” alleles; the quartile with the highest viral loads (>320,643 viral copies; mean = 971,587; median = 531,20) included 12 “bad” alleles. Interestingly, the distribution of predicted epitopes among HIV-1 proteins revealed that “good” HLA alleles focused more on Gag (Figure 2A) and less on Nef (Figure 2B). For “good” HLA alleles, predicted Gag epitopes increased 1.69 fold (p = 0.036) compared to the distribution found for “bad” HLA alleles, while predicted Nef epitopes decreased 2.35 fold (p = 0.038). When analyzed by individual protein, Gag- and Rev-specific repertoires showed more epitopes restricted by “good” HLA alleles than by “bad” ones, whereas there were more epitopes restricted by “bad” HLA alleles than by “good” ones in Nef, Env, Pol, Tat, Vif, Vpu, and also Vpr (albeit marginally) (Figure 2C). Nef- and Gag-specific epitope repertoires showed similar percentages of epitopes restricted by “good” alleles, however, the proportion of epitopes restricted by “bad” alleles was significantly higher in Nef compared to its proportion in Gag.

## Discussion

We systematically examined the immunogenic potential of HIV-1 at the population level through *in silico* estimation of the epitope repertoire of 302 HIV-1 proteomes. The number of predicted HIV-1 epitopes per proteome varied considerably between HLA alleles and thereby among individuals. Additionally, there were more epitopes identified in subtype B viruses than in subtype C, reflecting the existing bias of databases for inclusion of data from subtype B viruses and subtype B-infected individuals. Importantly, while we demonstrated that our analysis was not confounded by this experimental bias, it also highlights the need for better characterization of CTL responses against HIV-1 subtype C in the affected population (i.e., with typical motifs and HLA allele restrictions). Nonetheless, limitations to epitope prediction analyses intrinsically include biases derived from their training datasets, the fact that certain epitopes are not optimally defined or have incorrect HLA alleles restrictions (e.g., due to linkage disequilibrium) and pervasive of HLA class I allele promiscuity [45]. Despite those potential shortcomings, our findings corroborate those from immunological studies in this cohort. Principally, individuals with high viral loads tended to target preferentially Env and Accesssory/Regulatory proteins [18], [27], [44], [46], while individuals with low viral loads tended to make strong CTL responses against Gag [18], [27], [44], [46]. Additionally, by comparison with subtype B infected individuals Frahm and colleagues showed the importance of subdominant CTL responses for the control of replication in subtype C infected individuals [18], [27], [44], [46]. Collectively, those studies lend support to our *in silico* approach, especially in the context of a relatively limited knowledge of CTL responses in HIV-1 subtype C infection.

We also explored whether specificities of the epitope repertoires could affect clinical markers of disease progression. By integrating HIV-1 proteome-wide epitope mining to clinical and laboratory data in a South African cohort, our data showed a trend indicating that the number of HLA/epitope pairs was correlated both negatively with viral loads and positively with CD4 counts. Hence, HLA alleles associated with lower viral load in this cohort, referred to as “good” alleles, tended to present larger predicted epitope repertoires, than HLA alleles associated with high viremias, the “bad” alleles. This suggests that the inherent ability to present more epitopes could be a contributing factor to better clinical disease status. Alternatively, certain sets of epitopes may be needed to control the infection and thus, the more epitope motifs presented, the more likely individuals are to cover those epitopes. Our data alludes to a mechanistic paradigm in the cell-mediated immune response, supporting the intuitive assertion that control of HIV infection would capitalize on a broad repertoire while control would be stymied by a narrower epitopic pool. However, a nettlesome HIV characteristic is that despite eliciting relatively broad CTL responses, this generally does not result in the containment of the virus. Although attempts to correlate the breadth of the CTL antiviral response and control of HIV-1 infection *in vivo* have been equivocal [1], [2], [3], [4], [5], [6], [7], [8], [9], [18], it could nonetheless be beneficial for the host to have a larger epitopic pool to choose from – maybe not as a means to broaden the CTL response but rather to increase the probability of producing the more limited, effective set of CTL responses, since a diverse panoply of epitopes can be available for CTL recognition simultaneously and/or successively.

While the efficacy of the CTL response does not appear to rely solely on its breadth, it is widely believed that CTL escape has a major impact on disease outcome. As such, the limited epitope repertoire we identified for individuals/alleles associated with high viremia could reflect escape mutations that eliminated binding motifs from the autologous viral sequences.

In addition to quantitative distinctions, there were also qualitative differences between epitope repertoires restricted by specific HLA alleles: Those associated with better control of HIV replication were likely to present more Gag epitopes in their repertoire than “bad” alleles did; “bad” alleles were instead associated with a higher proportion of Nef epitopes. Interestingly, a recent study by Kiepiela and colleagues showed that Nef-specific CTL responses were associated with higher viral loads, unlike Gag-specific CTL responses, which were associated with lower viral burdens [2], [44]. While numerous reports have emphasized that CTL responses targeting Gag are the most tightly associated with the control of HIV replication [2], [10], [11], [12], [13], [14], [15], [16], [17], little is known about the underlying mechanism. Our study indicates that “good” alleles preferentially target Gag, and that within Gag there is an over-representation of epitopes restricted by “good” alleles instead of “bad” ones, as seen for all other HIV-1 proteins (except Rev). Interestingly, our results using clinical and laboratory data from infected individuals agrees with a very recent *in silico* study showing that HLA alleles with a low Relative Hazard (RH) of disease progression preferentially presented p24 epitopes [47]. Thus, discordant viral loads depending on specific protein targeting are apparently associated with particular HLA allele restriction sets for each protein. Nonetheless, this leaves open the question of what accounts for the beneficial effect on viremia: CTL responses focusing specifically on Gag, or CTL responses restricted by certain “good” alleles, or both.

The potential shortcomings of *in silico* epitope predictions cannot be entirely dismissed. And, notwithstanding the composite aspect of the cell-mediated immune response and the difficulty in ascertaining the relative importance of each attribute, evidence that the CTL response is in part mechanically predetermined could be significant in on-going efforts to define more palatable criteria of the immune response to assist vaccine design. Our findings are therefore relevant for vaccine design as they suggest the need to 1) maximize the number of possible epitopes to include in a vaccine candidate and to 2) direct the immune response toward Gag rather than Nef proteins [44], [48], [49].

## Materials and Methods

### Dataset

We evaluated 302 HIV-1 full-length plasma-derived genome sequences along with the HLA genotypes of the infected individuals. 270 subjects were from Durban (South Africa) infected with HIV-1 subtype C [38], [39] and 32 subjects from the Seattle PIC cohort (USA)[43] infected with HIV-1 subtype B [32], [40], [41], [42](and unpublished). Immunological and clinical data (viral loads and CD4 counts) were available at the time of virus sampling for a subgroup of the Durban cohort; details were described elsewhere [44]. HIV-1 amino acid sequences were derived for all recognized protein coding sequences of the 302 HIV-1 genomes. HLA allele frequencies in different ethnicities were obtained at http://www.ncbi.nlm.nih.gov/projects/mhc/ihwg.cgicmdPRJOVID9.

### Epitope Prediction

We employed an implementation of our previously described model [37] that uses logistic regression and leverages data across HLA alleles to predict CTL epitopes (http://atom.research.microsoft.com/hlabinding/hlabinding.aspx). The predictor was trained on all T-cell epitope data from the LANL [50] and IEDB (http://www.immuneepitope.org/home.do) databases in July 2006. Examples of non-epitopes (nine for each positive example) were obtained by randomly sampling proteins from UniProt [51]. Eight-, nine-, ten-, and eleven-mer predictors were trained separately. The prior probability of an epitope for each allele was set to 0.1. The prior probability of an epitope for a given allele of length *k* was proportional to the number of positive examples found for that length-allele combination in the datasets. A peptide-HLA pair was deemed a potential epitope if its posterior probability according to the predictor was greater than 0.5.

### Statistical analysis

Statistical analyses were done using JMP® version 5.1.2. Relationships between 2 variables were analyzed using Spearman's correlation factor Rho. Parametric Student's t tests were used to compare each pair of means.

## References

[1] Frahm N, Korber BT, Adams CM, Szinger JJ, Draenert R (2004). Consistent cytotoxic-T-lymphocyte targeting of immunodominant regions in human immunodeficiency virus across multiple ethnicities.. J Virol.

[2] Zuniga R, Lucchetti A, Galvan P, Sanchez S, Sanchez C (2006). Relative dominance of Gag p24-specific cytotoxic T lymphocytes is associated with human immunodeficiency virus control.. J Virol.

[3] Masemola A, Mashishi T, Khoury G, Mohube P, Mokgotho P (2004). Hierarchical targeting of subtype C human immunodeficiency virus type 1 proteins by CD8+ T cells: correlation with viral load.. J Virol.

[4] Gea-Banacloche JC, Migueles SA, Martino L, Shupert WL, McNeil AC (2000). Maintenance of large numbers of virus-specific CD8+ T cells in HIV-infected progressors and long-term nonprogressors.. J Immunol.

[5] Edwards BH, Bansal A, Sabbaj S, Bakari J, Mulligan MJ (2002). Magnitude of functional CD8+ T-cell responses to the gag protein of human immunodeficiency virus type 1 correlates inversely with viral load in plasma.. J Virol.

[6] Betts MR, Ambrozak DR, Douek DC, Bonhoeffer S, Brenchley JM (2001). Analysis of total human immunodeficiency virus (HIV)-specific CD4(+) and CD8(+) T-cell responses: relationship to viral load in untreated HIV infection.. J Virol.

[7] Addo MM, Yu XG, Rathod A, Cohen D, Eldridge RL (2003). Comprehensive epitope analysis of human immunodeficiency virus type 1 (HIV-1)-specific T-cell responses directed against the entire expressed HIV-1 genome demonstrate broadly directed responses, but no correlation to viral load.. J Virol.

[8] Musey L, Hughes J, Schacker T, Shea T, Corey L (1997). Cytotoxic-T-cell responses, viral load, and disease progression in early human immunodeficiency virus type 1 infection.. N Engl J Med.

[9] Liu Y, McNevin J, Zhao H, Tebit DM, McSweyn M (2007). Evolution of HIV-1 CTL epitopes: Fitness-Balanced Escape.. ePub.

[10] Novitsky V, Gilbert P, Peter T, McLane MF, Gaolekwe S (2003). Association between virus-specific T-cell responses and plasma viral load in human immunodeficiency virus type 1 subtype C infection.. J Virol.

[11] Ogg GS, Jin X, Bonhoeffer S, Dunbar PR, Nowak MA (1998). Quantitation of HIV-1-specific T lymphocytes and plasma viral load of viral RNA.. Science.

[12] Riviere Y, McChesney MB, Porrot F, Tanneau-Salvadori F, Sansonetti P (1995). Gag-specific cytotoxic responses to HIV type 1 are associated with a decreased risk of progression to AIDS-related complex or AIDS.. AIDS Res Hum Retroviruses.

[13] Geldmacher C, Currier JR, Herrmann E, Haule A, Kuta E (2007). CD8 T-cell recognition of multiple epitopes within specific Gag regions is associated with maintenance of a low steady-state viremia in human immunodeficiency virus type 1-seropositive patients.. J Virol.

[14] Sacha JB, Chung C, Rakasz EG, Spencer SP, Jonas AK (2007). Gag-specific CD8+ T lymphocytes recognize infected cells before AIDS-virus integration and viral protein expression.. J Immunol.

[15] Ramduth D, Chetty P, Mngquandaniso NC, Nene N, Harlow JD (2005). Differential immunogenicity of HIV-1 clade C proteins in eliciting CD8+ and CD4+ cell responses.. J Infect Dis.

[16] Crawford H, Prado JG, Leslie A, Hue S, Honeyborne I (2007). Compensatory Mutation Partially Restores Fitness and Delays Reversion of Escape Mutation within the Immunodominant Hla-B*5703-Restricted Gag Epitope in Chronic Hiv-1 Infection.. J Virol.

[17] Streeck H, Schweighardt B, Jessen H, Allgaier RL, Wrin T (2007). Loss of HIV-1-specific T-cell responses associated with very rapid HIV-1 disease progression.. Aids.

[18] Honeyborne I, Prendergast A, Pereyra F, Leslie A, Crawford H (2007). Control of human immunodeficiency virus type 1 is associated with HLA-B*13 and targeting of multiple gag-specific CD8+ T-cell epitopes.. J Virol.

[19] Carrington M, O'Brien SJ (2003). The influence of HLA genotype on AIDS.. Annu Rev Med.

[20] Migueles SA, Sabbaghian MS, Shupert WL, Bettinotti MP, Marincola FM (2000). HLA B*5701 is highly associated with restriction of virus replication in a subgroup of HIV-infected long term nonprogressors.. Proc Natl Acad Sci U S A.

[21] Trachtenberg E, Korber B, Sollars C, Kepler TB, Hraber PT (2003). Advantage of rare HLA supertype in HIV disease progression.. Nat Med.

[22] O'Brien SJ, Gao X, Carrington M (2001). HLA and AIDS: a cautionary tale.. Trends Mol Med.

[23] Gao X, Nelson GW, Karacki P, Martin MP, Phair J (2001). Effect of a single amino acid change in MHC class I molecules on the rate of progression to AIDS.. N Engl J Med.

[24] Carrington M, Nelson GW, Martin MP, Kissner T, Vlahov D (1999). HLA and HIV-1: heterozygote advantage and B*35-Cw*04 disadvantage [see comments].. Science.

[25] Kiepiela P, Leslie AJ, Honeyborne I, Ramduth D, Thobakgale C (2004). Dominant influence of HLA-B in mediating the potential co-evolution of HIV and HLA.. Nature.

[26] Frahm N, Adams S, Kiepiela P, Linde CH, Hewitt HS (2005). HLA-B63 presents HLA-B57/B58-restricted cytotoxic T-lymphocyte epitopes and is associated with low human immunodeficiency virus load.. J Virol.

[27] Frahm N, Kiepiela P, Adams S, Linde CH, Hewitt HS (2006). Control of human immunodeficiency virus replication by cytotoxic T lymphocytes targeting subdominant epitopes.. Nat Immunol.

[28] MacDonald KS, Embree JE, Nagelkerke NJ, Castillo J, Ramhadin S (2001). The HLA A2/6802 supertype is associated with reduced risk of perinatal human immunodeficiency virus type 1 transmission.. J Infect Dis.

[29] MacDonald KS, Fowke KR, Kimani J, Dunand VA, Nagelkerke NJ (2000). Influence of HLA supertypes on susceptibility and resistance to human immunodeficiency virus type 1 infection.. J Infect Dis.

[30] Jones NA, Wei X, Flower DR, Wong M, Michor F (2004). Determinants of human immunodeficiency virus type 1 escape from the primary CD8+ cytotoxic T lymphocyte response.. J Exp Med.

[31] Allen TM, Altfeld M, Geer SC, Kalife ET, Moore C (2005). Selective escape from CD8+ T-cell responses represents a major driving force of human immunodeficiency virus type 1 (HIV-1) sequence diversity and reveals constraints on HIV-1 evolution.. J Virol.

[32] Liu Y, McNevin J, Cao J, Zhao H, Genowati I (2006). Selection on the human immunodeficiency virus type 1 proteome following primary infection.. J Virol.

[33] Suhrbier A, Schmidt C, Fernan A (1993). Prediction of an HLA B8-restricted influenza epitope by motif.. Immunology.

[34] Larsen MV, Lundegaard C, Lamberth K, Buus S, Brunak S (2005). An integrative approach to CTL epitope prediction: a combined algorithm integrating MHC class I binding, TAP transport efficiency, and proteasomal cleavage predictions.. Eur J Immunol.

[35] Lundegaard C, Nielsen M, Lund O (2006). The validity of predicted T-cell epitopes.. Trends Biotechnol.

[36] Louzoun Y, Vider T, Weigert M (2006). T-cell epitope repertoire as predicted from human and viral genomes.. Mol Immunol.

[37] Heckerman D, Kadie C, Listgarten J (2006). Leveraging information across HLA alleles/supertypes improves epitope prediction..

[38] Rousseau C, Birditt BA, McKay AR, Stoddard JN, Lee TC (2006). Large-scale amplification, cloning and sequencing of near full-length HIV-1 subtype C genomes.. J Virol Methods.

[39] Rousseau CM, Learn GH, Bhattacharya T, Nickle DC, Heckerman D (2007). Extensive Intra-subtype Recombination in South African HIV-1 Subtype C Infections.. J Virol.

[40] Liu SL, Schacker T, Musey L, Shriner D, McElrath MJ (1997). Divergent patterns of progression to AIDS after infection from the same source: human immunodeficiency virus type 1 evolution and antiviral responses.. J Virol.

[41] Learn GH, Muthui D, Brodie SJ, Zhu T, Diem K (2002). Virus population homogenization following acute human immunodeficiency virus type 1 infection.. J Virol.

[42] Truong HM, Berrey MM, Shea T, Diem K, Corey L (2002). Concordance between HIV source partner identification and molecular confirmation in acute retroviral syndrome.. J Acquir Immune Defic Syndr.

[43] Schacker T, Collier AC, Hughes J, Shea T, Corey L (1996). Clinical and epidemiologic features of primary HIV infection.. Ann Intern Med.

[44] Kiepiela P, Ngumbela K, Thobakgale C, Ramduth D, Honeyborne I (2007). CD8+ T-cell responses to different HIV proteins have discordant associations with viral load.. Nat Med.

[45] Frahm N, Yusim K, Suscovich TJ, Adams S, Sidney J (2007). Extensive HLA class I allele promiscuity among viral CTL epitopes.. Eur J Immunol.

[46] Leslie A, Price DA, Mkhize P, Bishop K, Rathod A (2006). Differential selection pressure exerted on HIV by CTL targeting identical epitopes but restricted by distinct HLA alleles from the same HLA supertype.. J Immunol.

[47] Borghans JA, Molgaard A, de Boer RJ, Kesmir C (2007). HLA Alleles Associated with Slow Progression to AIDS Truly Prefer to Present HIV-1 p24.. PLoS ONE.

[48] Rolland M, Nickle DC, Mullins JI (2007). A new, core elements approach to vaccine immunogen design.. PLoS Pathogens: In press.

[49] Altfeld M, Allen TM (2006). Hitting HIV where it hurts: an alternative approach to HIV vaccine design.. Trends Immunol.

[50] Korber BTM, Brander C, Haynes BF, Koup R, Moore JP (2005). HIV Molecular Immunology 2005..

[51] Wu CH, Apweiler R, Bairoch A, Natale DA, Barker WC (2006). The Universal Protein Resource (UniProt): an expanding universe of protein information.. Nucleic Acids Res.

